# The Validity and Reliability of Characterizing Epilepsy Based on an External Review of Medical Records

**DOI:** 10.4178/epih/e2013006

**Published:** 2013-08-23

**Authors:** Bong Su Kang, Hae-Kwan Cheong, Ki-Young Jung, Sang Hyeon Jang, Jae Kook Yoo, Dong Wook Kim, Soo-Eun Chung, Seo-Young Lee

**Affiliations:** 1 Department of Neurology, Seoul National University Hospital, Seoul, Korea.; 2 Department of Social and Preventive Medicine, Sungkyunkwan University School of Medicine, Suwon, Korea.; 3 Department of Neurology, Korea University Medical Center, Korea University College of Medicine, Seoul, Korea.; 4 Department of Neurology, Eulji University College of Medicine, Daejeon, Korea.; 5 Department of Neurology, Konkuk University School of Medicine, Seoul, Korea.; 6 Department of Neurology, Kangwon National University School of Medicine, Chuncheon, Korea.

**Keywords:** Validity, Epilepsy, Epidemiology, Sensitivity, Specificity, Reliability

## Abstract

**OBJECTIVES:**

Our goal is to validate diagnosing and characterizing epilepsy based on a medical record survey by external reviewers.

**METHODS:**

We reviewed medical records from 80 patients who received antiepileptic drugs in 2009 at two hospitals. The study consisted of two steps; data abstraction by certified health record administrators and then verification by the investigators. The gold standard was the results of the survey performed by the epileptologists from their own hospital.

**RESULTS:**

The specificity was more than 90.0% for diagnosis and activity, and for new-onset seizures. The sensitivity was 97.0% or more for diagnosis and activity and 66.7-75.0% for new-onset epilepsy. This method accurately classified epileptic syndromes in 90.2-92.9% of patients, causes in 85.4-92.7%, and age of onset in 78.0-81.0%. Kappa statistics for inter-rater reliability and test-retest reliability ranged from 0.641-0.975, which means substantial to near-perfect agreement in all items.

**CONCLUSIONS:**

Our data suggest that epilepsy can be well identified by external review of medical records. This method may be useful as a basis for large-scale epidemiological research.

## INTRODUCTION

The Korean National Health Insurance (NHI) has provided health care for the entire Korean population as well as all medical facilities since 1989 and its database is a useful source of data for epidemiological research. Our previous study demonstrated its value in estimating the national prevalence of epilepsy based on antiepileptic drugs (AEDs) prescribed and diagnostic codes for claims [1]. However, NHI data should be validated for epidemiological research [2,3]. In addition, the NHI data do not provide detailed clinical information. Therefore, we launched the Epidemiological Study of Seizure and Epilepsy using Nationwide database for Corean Epilepsy patients (ESSENCE) project to estimate the prevalence of epilepsy from NHI data, which were validated and supplemented by a review of medical records.

Surveys reviewing medical records hold an advantage over door-to-door surveys, as they prevent recall errors, and clinical details as well as laboratory results are readily available [4]. When easy access to care providers is guaranteed in a national health care system, a review of medical records can be utilized in understanding the epidemiology of certain disorders. To ensure efficiency and consistency of the overall study, the task of reviewing medical records was carried out by trained external reviewers. The goal of this study was to develop a protocol for and evaluate the validity and reliability of medical record survey for epilepsy by external reviewers, to support our future epidemiological study.

## MATERIALS AND METHODS

### Subjects

The two study hospitals, Korea University Hospital (K hospital located in Seoul) and Eulji University Hospital (E hospital located in a suburban area), are both tertiary centers. K hospital has an electronic medical record system, whereas E hospital does not.

Among those who were prescribed AEDs during the year 2009, 80 patients were randomly selected from the NHI claims data from both hospitals. To guarantee an adequate representation of various conditions, for each hospital, we sampled 18 patients coded as having epilepsy or seizure, 12 as having central nervous system (CNS) illness other than epilepsy or seizure, and 10 without any diagnostic codes related to CNS disease. AEDs included carbamazepine, clobazam, ethosuximide, gabapentin, lamotrigine, levetiracetam, oxcarbazepine, phenobarbital, phenytoin, pregabalin, topiramate, vigabatrin, valproate, and zonisamide. Clonazepam was excluded because it is rarely used as monotherapy for epilepsy and is more frequently used for non-epileptic purposes. Other anticonvulsants, including primidone, felbamate and tiagabin were not available in 2009 in Korea. The diagnostic codes indicating epilepsy or seizure included G40* (epilepsy), G41* (status epilepticus), F803 (Landau-Kleffner syndrome), and R56.8 (convulsion), based on the 10th version of the International Classification of Diseases (ICD-10) and related health problems [5]. This study was approved by the Institutional Review Board of Korea University Hospital (AN10221-001).

### Procedures

The investigators developed a case recording form (CRF) and common diagnostic algorithm, which was tested for consistency. The CRF consisted of two parts: the first part was a preliminary form for chart abstraction, written in layman terms and included guidelines for surveyors; the second part was the verification form, which included the diagnostic algorithm for epileptologists (Appendix).

Part one documented demographics, ICD-10 codes, department of primary physician, diagnosis, antiepileptic drugs prescribed, history of seizures, recurrence of seizures, age of onset, new-onset seizure or presence of any seizure during 2009, description of seizure type, cause of epilepsy, electroencephalography (EEG) and brain imaging results. Age of onset was classified as <12 months, 12 months-6 years, 6 years-12 years, 12 years-18 years, 18 years-30 years, 30 years-60 years, and >60 years. EEG findings were categorized as normal, abnormal with focal epileptiform discharges, abnormal with generalized epileptiform discharges, and abnormal with non-epileptiform discharges.

Part two consisted of diagnosis, activity of epilepsy, cause, and classification of epilepsy. Diagnoses were categorized as: 1) epilepsy; 2) single seizure; 3) either epilepsy or seizure, unclear; 4) non-epileptic; and 5) either epileptic or non-epileptic, unclear. Epilepsy was defined as having two or more seizures during the patient's lifetime. In cases where it was uncertain whether he or she had single seizure or recurrent seizures, category 3 was assigned. Acute symptomatic seizures were categorized as 3, even if they were recurrent. If the AED was being used for other identifiable reasons such as pain, the patients were classified as being category 4. If the reason for prescribing an AED could not be determined, category 5 was assigned. Active epilepsy was defined as one or more seizures during 2009. The etiology was determined based on the clinical history, findings, brain imaging results, and EEG. If there were conflicting data, the etiology was determined by the clarity of the records and additional explanation from the treating physician. Non-specific imaging findings, such as small vessel disease, arachnoid cysts, venous anomalies, or diffuse atrophy were not considered as causes of epilepsy. Epilepsy was classified as: 1) generalized; 2) localization-related; 3) undetermined as to whether focal or generalized; 4) special syndrome, according to International League Against Epilepsy classification [6]; 5) and a lack of information for classification, based on the seizure type, syndrome diagnosis documented by the clinician, brain imaging, and EEG in the order of priority. Patients who had only generalized tonic-clonic seizures (GTCSs) with normal EEG and brain imaging were classified as 3. Patients who had only GTCSs with normal EEG but without any brain imaging results were classified as 5.

For chart abstraction, we recruited certified health record administrators (HRAs) who were experienced in reviewing medical records for epidemiological studies and health registry. These were coders certified by the Department of Ministry and Health Care of Korea for abstracting and managing data from medical records. The HRAs were intensively trained to review medical records and extract data related to epilepsy for 8 hours. The didactic portion included an overall introduction, a general overview on epilepsy, medical terminology and abbreviations commonly used in physicians' notes, how to find test results, and other conditions in the differential diagnosis or that are treated with AEDs. The HRAs then received hands-on training in completing the CRFs while receiving feedback from the epileptologists, until they achieved a high level of concordance with the epileptologists. Training was provided at a third-party hospital.

**Figure 1:**
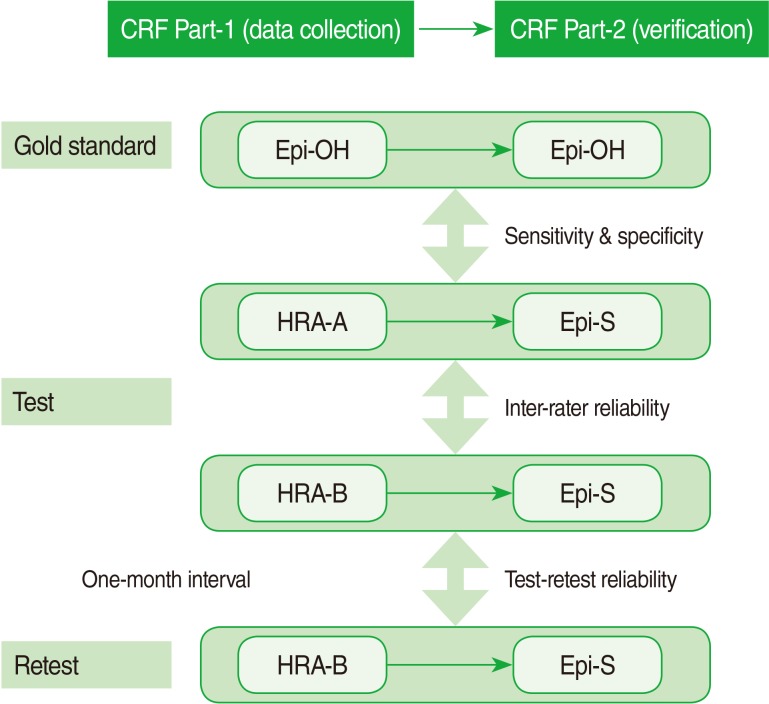
Study design for validation of medical record survey. The survey consisted of two-step data collection by certified health record administrators (HRAs) and then verification by the study epileptologist (Epi-S). For validation purposes, the gold standard was the conclusion each epileptologist made for his own hospital (Epi-OH), E or K hospital. In order to determine inter-rater reliability, two HRAs (HRA-A and HRA-B) reviewed medical records of the same patient while being blinded to each other. HRA-A repeated the reviews at a 1-month interval to determine test-retest reliability. CRF, case record form; HRA-A or B, health record administrator A or B; Epi-OH, epileptologist from the hospital; Epi-S, study epileptologist.

In this study, we assessed the validity and reliability of reviews performed by two HRAs (HRA-A and B), followed by verification by an external epileptologist (included SY Lee). HRA-A repeated the review at a 1-month interval to determine test-retest reliability. To establish a gold standard, each epileptologist performed both steps of chart abstraction and verification at his or her own hospital (E hospital, K hospital). In addition, alternative sources, such as the individual hospital data for patients with epilepsy or physician opinions were incorporated into the gold standard. The design of the study is summarized in Figure 1.

We analyzed the validity and reliability of the six items: diagnosis, activity of epilepsy, cause, classification (all verified by the epileptologist), new-onset epilepsy, and age of onset (obtained by the HRAs without verification by the epileptologist). The validity of the survey was estimated by measuring sensitivity and specificity. For multiple-choice items, we estimated the rate of the correct diagnosis. The level of reliability was estimated by kappa value, and graded to almost perfect (κ=0.81-1.0), substantial (κ=0.61-0.80), moderate (κ=0.41-0.60), fair (κ=0.21-0.40), or slight (κ=0.0-0.2) agreement, according to the classification system suggested by Landis and Koch [7]. Statistical analysis was performed using SPSS version 18.0 (SPSS Inc., Chicago, IL, USA).

## RESULTS

### Baseline characteristics

Of the total 80 subjects, 46 (57.5%) were men and the mean age of subjects was 48.1 years old (range 9-88). Thirty-nine subjects had epilepsy, one had a single seizure, and two had seizure but it was unclear whether this recurred or not. Thirty-seven subjects received AEDs for non-epileptic causes; among them, 29 received AEDs for neuropathic pain, 4 for prophylaxis of seizure after brain insult or surgery, and the others for hemifacial spasm, facial nerve injury, oromandibular dyskinesia, or cramps. There was one case where the diagnosis was unclear (Table 1).

### Validity

The sensitivity, specificity, and reliability of each surveyor are summarized in Table 2. For the diagnosis of epilepsy, the sensitivity was 97.6% for HRA-A and 100% for HRA-B, and specificity was 94.9% for HRA-A and 97.4% for HRA-B. For the activity of epilepsy, sensitivity was 100.0% for HRA-A and 97.0% for HRA-B, and specificity was 100.0% for both. For new-onset epilepsy, specificity was 90.9% for HRA-A and 100.0% for HRA-B, whereas sensitivity was 75.0% for HRA-A and 66.7% for HRA-B. The age of onset was correctly identified in 78.0% by HRA-A and 81.0% by HRA-B. The cause and classification of epilepsy were correctly identified and classified in 85.4% and 90.2% of cases, respectively for HRA-A, and 92.7% and 92.9%, respectively for HRA-B.

Inter-rater reliability between HRA-A and B was almost perfect for the diagnosis (κ=0.907), disease activity (κ=0.932), and classification of epilepsy (κ=0.864). For new-onset epilepsy, age of onset, and cause of epilepsy, the reliability was substantial (κ=0.728, κ=0.753, and κ=0.792, respectively).

The reliability of repeated examination was almost perfect for the diagnosis of epilepsy (κ=0.975) and substantial for disease activity, new-onset epilepsy, age of onset, cause, and classification of epilepsy (κ=0.796, κ=0.641, κ=0.697, κ=0.693, and κ=0.787, respectively).

## DISCUSSION

Our medical record review method demonstrated high levels of validity and consistency for the assessment of diagnosis, activity, and classification of epilepsy. The validity and reliability with regard to new-onset epilepsy, age of onset, and cause were acceptable.

The source of data from epidemiological studies may originate from direct population surveys, information or registries from physicians, medical records, or administrative data. Direct population surveys usually also involve interviews by non-physician surveyors or self-recorded questionnaires [8,9]. Some studies have validated questionnaires as a method to diagnose patients with epilepsy [8,9].

Reviewing medical records is highly useful to validate and supplement information from administrative data [8]. Reviewing of medical records is often performed by non-physician reviewers such as nurses or medical students, with or without a specialist's verification in epidemiological studies for epilepsy [9-11]. It would be ideal for the treating physicians to survey their own patients. However, not all of them are epileptologists and differences in experience would affect the consistency of the overall data. Although a standard protocol to confirm the diagnosis of epilepsy via medical records for epidemiological studies is not available, we are unaware of any other studies that have validated their tools. We showed that our non-physician reviewers could collect data that were sufficient for the epileptologists to diagnose epilepsy.

It is one of the limitations of our study that the gold standard was derived from medical records, lacking a face-to-face interview. We could not conclude which factors affected the validity, such as the type of medical record system or institution, specialties of physicians prescribing AEDs, or diagnostic codes, due to the small sample size.

Our results suggest that trained health care workers' review of medical records followed by verification by an epileptologist is a valid and consistent way to identify and characterize epilepsy patients. This allows large-scale epidemiological studies involving multiple distant locations to be performed at relatively low costs.

## Figures and Tables

**Table 1:**
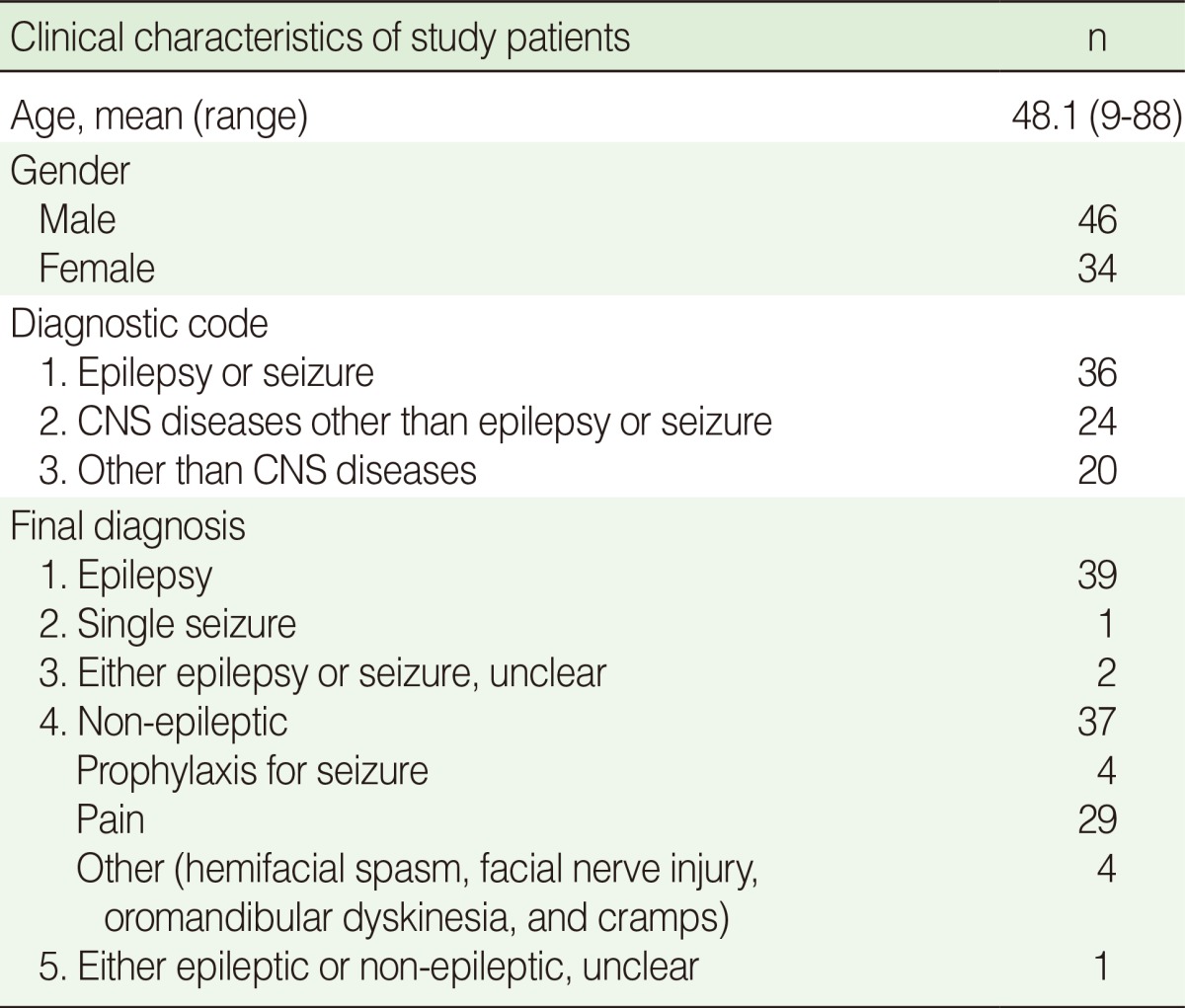
Patient demographics (n=80)

CNS, central nervous system.

**Table 2:**
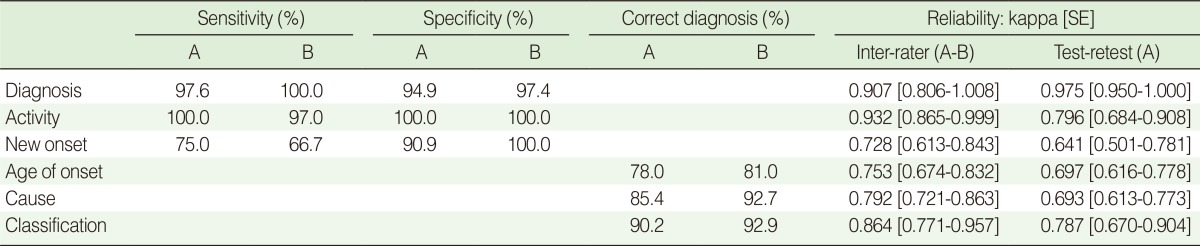
Validity and reliability of the survey

A, Health record administrator (HRA)-A; B, HRA-B; SE, standard error.
